
JOURNAL INFORMATION
==============================
NLM Title Abbreviation: Med Chir Rev
NLM Journal ID: 0034617

The Medico-Chirurgical Review

Publisher: Biomedical Journal Digitization Project

ARTICLE INFORMATION
==============================
PMCID: PMC4966269
PMID: 29917639
Article version: 1
Subjects: Reviews, Diseases Produced by Malaria

Observations on Hospital or Contagious Gangrene

Print publication date: 1828 Oct 1
Volume: 9
Issue: 18
First page: 323
Last page: 333
1828 Jul
License: This work is free of known copyright restrictions. For more information, please see PMC Back Issue Digitization.
License URL: https://creativecommons.org/publicdomain/mark/1.0/

==============================

experienced no bad symptoms whatever; twenty-nine were radically cured in from two to six months, and nine preserved, in spite of every thing, fistulous openings of greater or less extent, which obliged them to wear a bandage. Three-fourths of the anuses were the consequence of gangrene from strangulated hernia, and the other fourth of wounds, with loss of substance, in the intestine.

It appears, then, that the mortality of the operation is one in fourteen, and if we exclude the case of indigestion, which cannot fairly be considered a consequence of the operation at all, it is reduced to one in twenty.

We have thus, at considerable labour, presented our readers with an analysis of the above paper. In the original, it occupies nearly sixty pages, and we have compressed it, by means of a smaller type, and much abridgement, into ten or eleven. The memoir itself is interesting, the plan of treatment ingenious, and highly deserving of attention, as it promises, in many cases, to remove one of the most loathsome maladies that flesh is heir to.

---

### V.

*Observations on Hospital Gangrene, &c.* By J. Boggie, M.D. Surgeon to the Forces.

[Transactions of the Medico-Chirurgical Society of Edinburgh, Vol. III. Part I.]

Although names are conventional, and, in their etymology, frequently convey no idea of the things they were designed to express, it does not seldom occur that, by their misapplication, they either communicate one entirely, or partially, false. It would certainly be difficult to construct a vocabulary, every term of which would be an epitomized commentary upon the object to which it was appropriated; but we conceive that much improvement is required, and that much improvement could be made; and that, in those cases wherein it might prove impracticable to make names the etymological signs of things, it would at least be easy to select such appellations as would not mislead, when they could not instruct.

Hospital gangrene only denotes one variety of this disease, and a variety in its geography, not in its essence. But hospitals do not occupy the whole map of this affection; its blight visits different and distant localities, and no wound lies without the limits of its influence. Wherever its causes exist and operate, whether in the public lazar-house, or private home, its destructive properties will be seen and felt, and hospitals are merely the more frequent arenæ of its power, by being more frequently infested with the circumstances which favour its production.

We would, therefore, prefer the epithet *contagious gangrene*, as more expressive and not less correct. This designation conveys to us the leading feature of the disease; and, since the place of its occurrence is as accidental as the spots upon which pustules rise in a case of variola, we would discard an epithet too exclusive to be general, and too vague to be instructive. Dr. Boggie observes:

"It has, in this country, been denominated Contagious Gangrene, Phagedæna Gangrenosa, Malignant Ulcer, Putrid Ulcer, Sloughing Sore; and by the French writers, Gangrene humide des Hôpiteaux, Pourriture d'Hôpital: I shall use the name Hospital Gangrene, as a general term, and point out what I wish to be understood by that of Contagious Gangrene and Phagedæna Gangrenosa." 2.

Although it is not confined to any particular situation, its most aggravated forms are witnessed in hospital practice, from the more concentrated and active character of its exciting causes; and, whenever it appears in such unfavourable situations, the ravages it occasions are seldom equalled by any other local disease. When a wound or ulcer is seized with Dr. Boggie's first description of hospital gangrene—contagious gangrene, its sensibility is much increased, its healthy aspect gradually disappears, the granulations grow indistinct and flabby, vesicles sometimes form, purulent secretion ceases, and its surface becomes coated with a viscid brown crust. This coating soon cracks, and gives exit to a thin offensive ichor; an erysipelatous blush creeps over the sore, and often extends for a considerable way around it, the glands in its vicinity become tender and inflamed, the general surface is warm, the pulse quick and strong, the tongue furred, the bowels slow, and the patient is parched with constant thirst.

After the continuance of these symptoms for some time, the inflammation and fever gradually subside, or, becoming much aggravated, usher in the last stage, when blood generally begins to ooze from the sore, sphacelus takes place to a greater or less degree, the pulse sinks, the face becomes cadaverous, the sweat of death breaks upon the surface, and diarrhœa coming on quickly terminates the scene.

But, while this is the ordinary train of symptoms, the fever sometimes assumes a less phlogistic aspect.

"It has been often observed to partake more of a typhoid type; and it is of the utmost consequence in practice to attend to this distinction, as it will be found, that what would be a valuable remedy in the one case, might, if carried to any extent, be very pernicious in the other. The not attending sufficiently to this circumstance, that is, to the phlogistic or typhoid type of the fever, has, I am convinced, often led to fatal mistakes, and seems to be partly, at least, the cause of that great diversity of opinion among medical men regarding the best mode of treating this very dangerous affection." 6.

Dr. Boggie's second, or *phagedænic variety* of gangrene, seldom attacks a recent discontinuity of surface, is less vigorous in its action, and less rapid in its course. Its commencement is usually announced by the appearance of a small, dark-coloured ulceration on the edge of the sore, which is, in general, circular, and secretes a matter of peculiar smell. Other ulcerated patches soon appear, and, spreading slowly over the wounded surface, unite, and often kindle such constitutional irritation, as accelerates the progress of the local disease. The discharge becomes bloody and fetid, the ulceration often creeps over the margin of the sore, sphacelus not unfrequently occurs, and the patient before death is sometimes doomed to suffer the concluding stage of the preceding and more acute form of the disease.

Dr. Boggie's division of hospital gangrene into contagious and phagedænic gangrene seems rather objectionable. In a subsequent passage, the Doctor acknowledges the contagious nature of this disease, and, in a preceding extract, we have shewn that hospital gangrene is used by him as a general term, under which he delineates these two varieties. But, if the general disease be contagious, every species of that disease must be contagious; otherwise a genus may contain species which are destitute of the generic character, and species may exhibit properties and symptoms which do not enter into the constitution of the genus. To divide a disease, therefore, in itself contagious, into two varieties, one of which is distinguished from the other by this contagious property, is a *constructive refusal* of this property to the second variety, and is the appropriation of a generic or common symptom to an individual modification of the disease.

In mild cases of hospital gangrene, the parts chiefly involved are the skin and cellular membrane, but in more inveterate varieties, all the subjacent textures are successively attacked, so that, the soft parts being removed by sloughing, the bones are exposed, and often die.

"A great peculiarity in the phagedenic form of the disease is, that different actions, such as the ulcerating, suppurating, and cicatrising, may frequently be seen going on in one sore at the same time." 9.

Artery resists more successfully the ravages of gangrene than any other texture, yet it seldom happens that the secretions are unmixed with blood, and death has occasionally arisen from profuse and repeated hæmorrhage. "We have been obliged," says Delpech, (Mem. sur la Pourriture d'Hôpital, p. 22) to throw a ligature around the axillary artery, from an accident of this nature, which had involved the superior part of the humerus; and, on another occasion, a similar affection, which was accompanied by frightful disease of the entire arm, put me to the necessity of amputating the member." Specific sores are less obnoxious to gangrene than any other solutions of continuity; yet, that they are not always exempt, Dr. Hennen furnishes us with a very remarkable proof in the case of a soldier, who was sent into the hospital at Bilboa, with an open bubo in the groin which was almost instantly seized with gangrene, and who survived its attack only 48 hours.

Our author is very full on the causes of hospital gangrene, which he arranges under seven heads, and on the principal of which we will make a few observations.

It will be generally admitted that a fresh and uncrowded atmosphere conduces much to the preservation of health and removal of disease; but why close and ill-ventilated apartments favor the appearance of this peculiar affection, it is not easy to explain. It is seldom seen in hospitals, when the wards are thinly occupied and freely ventilated, and, when it does appear, it is seldom fatal, and seldom spreads; but in crowded apartments, and unhealthy districts, it is frequently met with, and proves, in general, severe. In such cases, the air is impure, and unable to impart that vital property to the blood, which is essential to health, and conducive to the cure of malignant ulcers. But, we apprehend that a contaminated atmosphere does not always act upon the sore through the medium of the system, but directly upon the sore itself. Many modern experiments have shewn the power which different gases exert upon malignant ulcers, and we think there can be no doubt, that the qualities of the external air will materially influence their course and condition. The noxious particles with which it is adulterated may act immediately as a poison upon the abraded surface, and, by being taken up by the absorbents, may blight the sore by contaminating the constitution.

But, in crowded ships, hospitals, and other receptacles of disease, the air is not only injurious from its impurity, it is insalubrious from its increased temperature.

"It is well known that in hot climates, in addition to the general excitement of the system, all the secretions and excretions are more acrid, and, in a very particular manner, the secretion from sores, which is both very abundant and extremely irritating. During the prevalence of the gangrene at Bilboa, and when it was at its very height of malignancy, of which so animated an account is given by Dr. Hennen, in his Principles of Military Surgery, p. 210, *et seq.*, the thermometer in the shade seldom stood below 75° of Fahrenheit, and often much higher. In the wards of the hospital I have seen it at 85°, and in the sun upwards of 120°, indeed, I know of no exception to this fact of hospital gangrene prevailing most in hot weather, for I have constantly found, that, in the warm months, the disease raged in its greatest virulence. I am, therefore, inclined to consider a greatly heated atmosphere to be one of the most powerfully exciting causes of this disease." p. 13.

*Inattention to cleanliness* is another very frequent cause of hospital gangrene. When the secretions of a sore are not carefully and regularly removed, they soon become acrid stimuli, both to the secreting surface and the surrounding texture, aggravate or induce inflammation, and favour the extension of the original disease. Dr. Boggie believes that its introduction into the Artillery hospital, at Woolwich, is only to be accounted for on this principle; and, indeed, Dr. Rollo admits, that very little care was taken of the wounds, from the singular conviction, that to wash them was superfluous, if it did not tend to increase the mischief.

Upon the same principle acrid applications have been occasionally suspected as the predisposing, or exciting cause of this disease. When ointments are largely used and irregularly renewed, they lose their mild and soothing properties, become rancid and irritating, and impede the cure which they were intended to effect. They have certainly been much too indiscriminately employed, and the success with which wounds and ulcers are at present treated, may be partly accounted for by the less frequent and more judicious application of such remedies.

*Intemperance in drink and diet, mental and mechanical irritation* will, always predispose to, and often produce hospital gangrene. The general character of this disease seems to be inflammatory, and it is only, we presume, when it assails constitutions much impaired by disease or age, that it exhibits a low or typhoid aspect. It is, therefore, obvious that any thing which encourages a phlogistic habit of body, under such circumstances, will add fuel to a complaint generally phlogistic, and nothing can more effectually induce such a habit than intemperance. Dr. Boggie's observations on this subject are so just, and of such importance, that we will not abridge his sentiments by transferring them into our own language.

"From an idea which very generally prevailed, that the accompanying fever was typhus, and that hospital gangrene could not be prevented, or successfully treated, unless by stimulants, antiseptics, and tonics, a liberal allowance of wine was made to every patient as a preventive; and, when the disease actually appeared, it was then prescribed in increased quantity as a cure; the consequence of which was, a fatal termination in almost every case." p. 19.

"The fever appeared to me, from the first, to be highly inflammatory, that is immediately after the injury was received, and at a time when the heat of the season was at its maximum; and that it became more mild when the temperature fell, or when the sedative effect of the cold began to be felt; but it was not until the antiphlogistic treatment was fully established, that any remarkable decrease in the mortality took place." p. 20.

Perhaps there is nothing in the cure of wounds and obstinate ulcers of more importance than bodily rest and mental quiet. The irritation caused by motion keeps the diseased parts in a continual state of morbid excitement, nature is interrupted in her ordinary proceedings, and an injury which was at first trifling and promised well, gives rise to a severe and protracted illness, which at last too often terminates in death.

"I shall never forget the appalling spectacles I beheld in the unfortunate men who effected their escape from Talavera; though the wounds of many of them were but slight, and, no doubt, would have soon got well had they remained at rest, and on the antiphlogistic regimen; yet, after a few days, in consequence of the motion, and other irritating causes to which they were subjected, they were to be seen in all stages of the disease, from the state of incipient inflammation, to that of gangrene, and even sphacelus, and loathsome from the quantity of maggots with which they were infested; and, in those men who arrived at Bilboa from Vittoria, many of them were in an equally bad state; whole limbs were to be seen, almost entirely deprived of vitality, nearly dropping off, and a prey also to vermin, which seemed actually to be devouring them." 30.

Whether hospital gangrene was ever propagated by contagion was once a disputed and doubtful question. But, since the days of Trotter, no author of distinction has adopted his doctrines, and it is now very generally believed, that the disorder is both contagious and infectious. Whilst Dr. Boggie, however, ranges himself among the Contagionists, he believes that more ought to be ascribed, during the epidemic spread of hospital gangrene, to the continuance and continued operation of the causes which first gave birth to it, than to any contagious power which it may possess; for it has sometimes occurred to him that—

"Although all the means which are usually employed for the extinction of contagion were had recourse to, such as fumigations of nitric and muriatic acid gases, ventilation, separation, nothing appeared to have the least effect in moderating the violence of the disease, till the antiphlogistic regimen was established." 25.

The principal facts in favour of its contagious nature are furnished us by Dr. J. Thomson, who has given in his *Lectures on Inflammation*, one of the fullest and most perfect descriptions of the disease; and, as they are very concisely stated, for the information of those who may not have access to his valuable works we will, no doubt, be excused for inserting them.

"The contagious nature of hospital gangrene appears to me to be sufficiently proved, 1*st*. By the fact that it may be communicated by sponges, charpie, bandages and clothing, to persons at a distance from those infected with it; 2*ndly*. By its having been observed to attack the slight wounds of surgeons, or their mates, who were employed in dressing infected persons; and that even in circumstances where the medical men so employed did not live in the same apartment with the infected. 3*dly*. By our being able often to trace its progress distinctly from a single individual through a succession of patients. 4*thly*. By its attacking recent wounds as well as old sores, and that in a short time after they are brought near to a patient affected with the disease. 5*thly*. By our being able to prevent the progress of disease in particular situations, by removing the infected person before the contagion which his sores emit has had time to operate. 6*thly*. By its continuing long in one particular ward of an hospital, or in one particular ship, without appearing in other wards or ships, if pains be taken to prevent intercourse between the infected and uninfected." *P*. 484.

We have now enumerated what our author considers the most frequent and active causes of hospital gangrene, and a moment's reflexion upon their character will convince us, that they are generally such as either directly produce, or materially favour inflammation. Most of them being active stimuli, will directly augment the action of the texture to which they are applied. Such are heat, irritating medicaments, motion, neglected secretions, and mental excitement. Polluted air and contagion may, probably, produce an opposite effect, or act as sedatives upon the sore, through the general system, or, immediately upon the sore itself. And thus can we very reasonably account for the two opposite characters which this disease occasionally assumes, and satisfactorily reconcile the different views which writers of equal acumen and experience have maintained respecting it. By some it has been deemed atonic and treated with stimulants; by others it has been regarded as inflammatory. Now, we believe, that both are equally correct, if we only attach a partial signification to their views. When we consider the principal causes of this disease, the symptoms of its first stage, and the remedies usually effective, there can be little doubt that hospital gangrene is, in the majority of instances, essentially inflammatory. But we are equally certain that it may appear, under peculiar circumstances, with very different features, and may, therefore, require a very different plan of management.

An attentive consideration of the causes of hospital gangrene will likewise assist in solving another question which has been much agitated;—in what relation do the local and constitutional symptoms stand, with a reference to their order of succession? Are the local symptoms first developed, and is the system subsequently involved; or does the constitutional disturbance give rise to the local disease? If gangrene succeed a cause purely local, such as irritating applications and mechanical disturbance, the systematic affection must be consecutive; but, if the attack can be traced to a defiled atmosphere, depressing passions, contagious miasmata, or such general causes as may operate primarily upon the constitution, we have then reason to conclude, that the general disorder is not the consequence, but the cause of the topical symptoms.

There cannot be much difficulty in drawing our diagnosis of a disease so prominently characterized.

"There is a species of gangrene complicated with a violent remittent fever, like the yellow fever, which may be mistaken for Hospital Gangrene, but is very different. It attacks wounded men, particularly after undergoing amputation, and has proved fatal I think in almost every instance that I have seen. The gangrene in this case appears to be symptomatic of the constitutional derangement." 47.

Larrey reports that it occurred very frequently after operations during the campaign in Egypt, and Dr. Hennen, in his Work on Military Surgery, has given a particular account of it. To state a few of its leading signs will be sufficient to distinguish it from the present affection. Some hours after the operation, it is ushered in with symptoms very similar to those of yellow fever; —rigor, succeeded by heat and perspiration, which follow each other at intervals of five or six hours—the skin soon becomes yellow, gangrenous spots soon appear upon the stump, and, coma coming on, the patient dies on the first or second day of the complaint. In addition to this disease, scorbutic ulcers are, perhaps, the only sores with which it can be confounded. But the absence, in Hospital Gangrene, of the general symptoms of scurvy, the presence of fever, the indifference which it manifests, in its selection of subjects, to the freshness or saltness of their provisions, and the inefficacy of a vegetable diet to remove it, are sufficient to constitute a line of demarcation between the two diseases.

"The prognosis in this disease, as in most others, must depend on circumstances. If the patient is young and healthy, of temperate habits, and the accompanying fever inflammatory, even although the affection should be pretty severe, the prognosis, I think, may be favourable; but, on the contrary, if the patient is old, addicted to intemperance, his health bad, and particularly, if the gangrene should be complicated with fractures of the bones, or a scorbutic diathesis be present, with a fever evidently typhoid, it must be very unfavourable." 32.

In consequence of the double series of symptoms in hospital gangrene, its treatment must, in general, be both constitutional and local; for, whether the sores have infected the constitution, or the constitution the sores, the topical affection, if neglected, will aggravate the general disturbance, and the general disturbance, if unattended to, will increase the local disease. In some cases, it is true, the attack is so mild, that the system may scarcely be said to sympathize, and, in such instances, our local remedies may alone affect a cure; but it is prudent to watch every avenue to danger, since, by proper precautions, we may frequently prevent what it might be found difficult to cure.

In young subjects, and those hitherto strong and healthy, Dr. B. has found the accompanying fever almost always inflammatory, requiring blood-letting, in proportion to its violence and the strength of the patient.

"No precise rule can be laid down regarding the quantity to be taken; but in severe cases I have had occasion to take one, two, three, or even four pounds of blood before the inflammatory symptoms were subdued. In men, however, of a less robust constitution, who may have lingered long in hospital, or suffered much from ill health, we must act with the greatest caution. Bloodletting in such, is either altogether inadmissible, or should be used very sparingly. The same may be said of those who are the subjects of Hospital Gangrene in civil hospitals. They are for the most part poor people, worn out with age, or whose constitutions have been broken by poverty and hard labour; in such the disease assumes a different character, and will require a corresponding treatment." 34.

Some have objected to the employment of the lancet, through fear of the punctures becoming gangrenous; but the Doctor has never seen a single instance of gangrene supervene upon the operation. Indeed, if blood-letting be had recourse to when blood-letting is required, we can scarcely conceive that such a consequence will often follow. It will generally occur, when the accompanying fever is typhoid, and the vital powers low; but, in such instances, the lancet is an improper remedy, and the danger of its employment will not mainly consist, we apprehend, in any symptoms that may arise from the puncture, but in the detraction of a fluid which was necessary to life. But others oppose venesection on a more important ground. They advocate the adynamic nature of the disease, and, consequently, their principal remedies are bark and opium, camphor, ammonia, and wine. If they ever extract blood, it is locally, or in trifling quantities. Such contrariety of principles and practice we have already accounted for, and, while we maintain the value of depletion under proper restrictions, we know of no author, so blind to symptoms and to circumstances, as to advocate the lancet indiscriminately and without distinction.

"It has already been said, that, in that form of Hospital Gangrene named Phagedæna, very great destruction of parts may sometimes take place, without the constitution being much affected by it. Now, if blood-letting is used in such a case, or even in the advanced stage of the true inflammatory gangrene, when disorganization has taken place, and the system is sinking under the consequent debility, the vital powers being nearly exhausted, the result must be obvious; but when it is used with caution, and in cases where it is really applicable, it will be found, I doubt not, to be a most valuable remedy." 36.

In importance, cathartics are only second to blood-letting, and may frequently be administered when the latter cannot be employed. If the patient have been long confined, or of a weakened habit, if the symptoms be of an inflammatory character, yet if the pulse be weak and easily compressed, brisk and repeated purging will sufficiently lessen the unnatural excitement, and, in those instances in which the fever is not purely phlogistic, but exhibits a mixed and undecided character, purgatives will prove the most useful, because the safest evacuants.

Ponteau has warmly advocated the importance of emetics in this disease, but they are, unquestionably, much inferior to cathartics, by being less generally applicable. When the chylopoietic organs are undisturbed, the object to be gained by giving an emetic is not very obvious. If the symptoms be inflammatory, venesection and cathartics will make a much more permanent impression on the disease, and, if they be asthenic, emetics will be more likely to augment debility than afford relief.

"The cases to which they are chiefly applicable, are those where the stomach is loaded, and where the fever appears to be of a bilious character. This was the case in not a few which I saw at Bilboa; the gangrene being complicated with a fever of that kind, and sometimes supervening to it. Emetics in nauseating doses, were often tried in such instances, and were considered useful, both by their operation as evacuants, and by the nausea which they excited, lowering the action of the vascular system. In the same cases, antimonials and saline diaphoretics will be found very useful." 37.

Every stimulating medicine is reprobated by our author in the first stage of this disease. Bark, opium, camphor, and wine, are well known excitants, and if hospital gangrene merits and meets the character we have drawn of it, stimuli must not be exhibited until inflammatory symptoms have subsided, and the disease have put on a more mild or chronic aspect. Let not this observation, however, be regarded as a rule. There may be and are many instances in which strengthening regimen and tonic remedies will be required from the first, and wherein the application of phlogistic measures would be hazardous, if not destructive. When the fever is from the beginning decidedly typhoid, when the patient is exhausted by protracted illness, or worn down by age, or when the irritation of the local disease is undermining the constitution, any of the above medicines may be given, and all may be necessary.

It is only when the patient is robust and the symptoms are inflammatory, when the pulse is strong and the heat is great, when foulness of tongue, loss of appetite, thirst, and watchfulness, announce the existence of no ordinary excitement, that all such stimulating medicines ought to be avoided. In obedience to the same views, the diet should be frugal, mild cooling diluents should be employed for drink; and the apartments of the sick should be freely ventilated. Some have recommended cold affusion, but as its sedative effects are soon removed by re-action, unless it be long continued, there may be some danger of its ultimately aggravating the disease. Sponging with tepid water is, perhaps, as soothing, and is a less doubtful application.

The Doctor has overlooked the mineral and vegetable acids in his list of general medicines, and, since they are not liable to the same objections with bark and wine, but may be used with equal safety in both forms of the disease, we deem the omission as of some importance. The sulphuric acid is most usually employed, but the oxymuriatic, citric, and tartaric acids, may be given with advantage, and their efficacy is, generally, the greater when the dose is considerable.

As to the treatment which should be pursued with a reference to the local symptoms, many plans have been proposed, and many remedies recommended. Indeed, the French esteem constitutional treatment nugatory, and look exclusively to local remedies for a cure. Delpech is particularly urgent on this side. He says that he had witnessed this disease during three destructive epidemics; that, from the facts then noticed, he became convinced that it was a purely local affection, arising from a local contagion; that all the attempts which were made to arrest its progress by constitutional treatment proved unavailing, and that topical remedies alone were found of use. But if it be essentially the result of contagion, is not contagion a general cause, and will it not act upon the general system? Delpech, to be consistent, believes that it only operates upon the surface of the sore, and that this circumstance accounts for the success of the actual cautery, which burns away the infected substance, and thus extinguishes the disease. But we know of no such local contagion, or infectious principle. The poison of the rattle-snake, or of a rabid animal, and the venereal, or small-pox virus, are purely local in their primary action, as local, assuredly, as the contagious matter of hospital gangrene; yet, the effects which they produce are not limited to the surface to which they were applied, and the medicines best calculated to remove these effects, are not such as are endowed with local virtues.

Many cases, we believe, from the nature of the causes producing them, are originally constitutional, ultimately involving the topical injury; and, to treat such cases locally, we apprehend, were just as judicious as would be our treatment in inoculated small-pox, if we confined our attention to the punctures of the lancet, or to the dressings of the pustules.

Our author arranges his topical applications into three classes; viz. sedatives, escharotics, and stimulants. Sedatives are best adapted to the first stage, and, perhaps, the application of cold will be found as effectual in alleviating, as it is in preventing the disease. For this purpose, cloths, moist with cold water, should be kept constantly applied; and, to increase their effect, artificial means may be had recourse to, to lower still further their temperature.

Poultices are very doubtful remedies in the first stage. If warm, they are stimulating and generally hurtful, and, if cold, the heat of the parts will soon make them warm. They are also objectionable on account of their weight. When the inflammatory symptoms have subsided, however, and when the sloughs are not disposed to separate, they may be often useful, accompanied with warm fomentations. Under such circumstances stimulants are necessary. Diluted, or concentrated acids, nitrate of silver, oil of turpentine, caustic potash, the arsenical solution, and actual cautery, may be advantageously applied. These should be assisted by a more generous diet and small quantities of wine. But the greatest caution is required in the use of stimuli, since excess, even to a small extent, will seldom fail to occasion a relapse, which is infinitely more dangerous than the first attack. When the sloughs separate, healthy secretion is established, and florid granulations rise, the wound should be dressed with dry lint, over which a pledget of mild ointment may be supported by a compress and roller. If the dressings be not regularly renewed, the discharge becomes acrid, irritates the healing surface, and retards the cure. The granulations get weak and inactive, and the phagedenic variety will be soon established.

This second form of hospital gangrene is of a more local nature than the first, and, if timely treated, may in general be arrested by local remedies, the most efficacious of which are the escharotics above enumerated.

The argentum nitratum and oxyd. hyd. rubr. are those which the Doctor has chiefly employed, but Mr. Welbank strongly recommends nitric acid, which is the application principally employed in St. Bartholomew's Hospital since the publication of his paper; Mr. Blackadder believes that the arsenical solution will cure almost every variety; and Dr. Rollo reports that the oxygenated muriatic acid was made use of in the Artillery Hospital of Woolwich with great success.

Speaking of the arsenical solution, the Dr. observes—

"At the time when this disease raged with so much violence at Bilboa, I had not heard of that remedy; and when I was made acquainted with it, the malady had abated so much, and was so manageable by the means then in use, that it was not thought necessary to have recourse to any other, and since that time I have had no opportunity of putting it to the test. That it is a valuable remedy in certain states of the disease, there cannot, I think, be a doubt; but that it will succeed in all, and without the assistance of general remedies, remains I conceive yet to be proved." 45.

The actual cautery, however, is the "magnum remedium" in France. Dupuytren seldom uses any other, with the exception of the lancet; and Delpech observes, that in his practice nothing equalled the prompt and invariable success attending it; a single application being, for the most part, sufficient, when the iron could be carried over every point of the diseased surface. It seems, however, to be too rough an application for British nerves, and, although many of our objections against its employment may be ascribed to prejudice rather than to reason, there is no immediate probability of its gaining that ascendancy in the therapeutics of England, which it enjoys in those of France. When milder measures are equally successful, it is worse than useless to add the frightfulness of terror to the pungency of pain, and to increase actual misery with useless fears.

"It has been said, that, in Hospital Gangrene, when all other means fail, amputation may be performed with success, and even at a time when the gangrene is extending.

"There is one form of Hospital Gangrene, which appears to be entirely local, at least in the commencement; and I have sometimes seen very great destruction done by it, without the constitution being much affected, viz. the phagedenic form of the disease; in which I have no doubt but that the operation may be performed, and without any risk of the disease returning, or affecting the stump; but in the other form of the disease, which has been named Contagious Gangrene, in which there is always inflammation to a greater or less degree with fever, I consider such practice to be extremely dangerous, and contrary to all the rules which have ever been laid down on this subject.

"The operation, in my opinion, should never be thought of, until the fever and inflammation abate, and then, in all probability, the progress of the gangrene will be found to be stopped." 49.

In that species of gangrene which is complicated with remittent fever, and to which we have alluded when speaking of the diagnosis, every mode of treatment hitherto tried has proved equally unavailing; death taking place in little more than 36 hours after the accession of the fever.

"It appeared to me most advisable, after prescribing an emetic, and cathartic, to support the strength of the patients as long as possible. With this view, bark was ordered, with wine and other cordials, and the stumps were dressed with a powder composed of myrrh, bark, and camphor, which I have sometimes applied with advantage to mortified parts: but nothing was of any avail; they all sunk in the short space of time already mentioned." 49.

Dr. Boggie's very valuable and instructive Essay concludes with three tabular views, illustrating the results of different modes of treatment, and confirming the orthodoxy of the preceding doctrine. While stimulants were exhibited the proportion of deaths to diseases treated was 1 to 15; but, when antiphlogistic measures were adopted, the proportion diminished to 1 to 34, and, so soon as they were exclusively followed, the mortality decreased to 1 in 132. But such successful results are not always to be expected from the most judicious measures. The experience of other writers, entertaining the same views with Dr. Boggie, has furnished us with very different bills of mortality; and we doubt not that, while some attacks have been less destructive than others, we should, in our general deductions, study the character of the epidemic, in estimating the success of its treatment.

In a paper of such circumscribed limits, it was impossible to introduce every point and discuss every question, which attaches to a subject of so much practical interest as Hospital Gangrene; many particulars have been, consequently, overlooked, and some have been imperfectly noticed; but the topics, which the author has considered it proper to enlarge upon, have received justice, and, in the course of our observations, we have occasionally endeavoured to supply the blanks with important extracts and original remarks.

---

### VI.

### *Memoir on Visceral Neuralgia.* By P. JOLLY, M. D.

Much has been written on that class of painful maladies, the neuralgiæ of the nerves of relation—and, under the class *neuroses*, many diseases, or rather symptoms, connected with or dependent on visceral neuralgia, have been accurately described, as for example, angina pectoris, spasmodic asthma, hooping-cough, &c. Few or no investigations, however, have been made into the state of the visceral nerves themselves, on which the disorders of function in these viscera, so often depend. The general sentiment, indeed, appears to be that, in the *neuroses*, there is a total absence of appreciable change of structure, or, in other words, of physical lesion, in the parts affected. Our author thinks, on the contrary, that there are very few of the neuroses, whether external or internal, in which some lesion might not be detected either in one of the principal nerves, their divisions, their anastomoses, or in one of the great nervous centres. The researches of Bichat, Beclard, Swan, and others, on the local affections of nerves, justifies him in this opinion, as far as the nerves of relation are concerned. These researches have reduced the number of vague terms in medicine, and augmented the number of those which have some anatomical and physiological meaning. But we have not been so successful in our researches respecting the visceral or ganglionic nerves. All there is darkness; and we have hardly ventured to apply the term *neuralgia*—probably because the idea of great pain is associated with the word; whereas, the splanchnic nerves, having specific kinds of sensibility, may suffer severely in their own way, and greatly disorder the functions of the organs on which they are distributed, without any sensible pain, in the common acceptation of the word.

M. Jolly employs the term NEURALGIA, then, to designate every lesion, physical or vital, direct or indirect, of a nerve, unaccompanied by external signs of inflammation—assuming, for the most part, a periodical character—and disturbing, more or less, the functions of the corresponding viscera. He reserves the term "NEUROSES," on the other hand, for all those nervous affections where no local cause can be traced in the nerve, but merely disorder of function in the organ. Our author properly observes that, between the cerebro-spinal nerves (the nerves of relation with the outward world) and the ganglionic nerves arising from the solar plexus, there is an intermediate class (the phrenic and eighth pair) which partake of the characters and functions of both the others—and whose disorders are modified accordingly. It is with this class of nerves that our author commences his history of the neuralgiæ of the nutritive life—or the function of digestion and assimilation.
